# Co-evolution of RcsD and RcsF shapes signal transduction in the enterobacteriaceae Rcs system

**DOI:** 10.1371/journal.pone.0358890

**Published:** 2026-09-25

**Authors:** Abdelraouf O. Dapour, Moustafa Shehata, Rania Siam, Nahla A. Hussein

**Affiliations:** 1 Biotechnology Graduate Program, School of Sciences and Engineering, The American University in Cairo, Cairo, Egypt; 2 Bioinformatics and Computational Biology Program, Iowa State University, Ames, Iowa, United States of America; 3 Modern Sciences and Arts Universities, Giza, Egypt; 4 Ross University School of Medicine, Saint Micheal, Barbados; 5 Molecular Biology Department, Biotechnology Research Institute, National Research Centre, Cairo, Egypt; Yeungnam University, KOREA, REPUBLIC OF

## Abstract

Two-component systems (TCS) formed of sensor histidine kinases (HK) and response regulators (RR) are the primary prokaryotic signal transduction pathways. In Enterobacteriaceae, some TCS evolved architectural complexity, by integrating auxiliary proteins into the HK– RR dyad. The Regulator of Capsule Synthesis (Rcs), a conserved phosphorelay system in Enterobacteriaceae, recruits several auxiliary proteins, including an outer membrane lipoprotein, RcsF, which senses cell envelope threats and transduces the signal to downstream components. A second auxiliary protein located in the inner membrane, RcsD, transfers the phosphoryl group from the hybrid histidine kinase RcsC to the response regulator RcsB. RcsD is formed of a large periplasmic domain (Asn43-Asn308) sandwiched between two transmembrane helices and a cytosolic domain. For RcsD, the role and evolutionary benefit of maintaining a large periplasmic domain while the main phosphostransfer reaction occurs in the cytosol is not clear. Here, we show that RcsF and RcsD exhibit strong co-evolutionary coupling independent of host speciation (partial Mantel r = 0.950, p < 0.0001). Structure-guided docking and 500-ns molecular dynamics simulations predicted a stable interaction between RcsF and RcsD (MM-GBSA ΔG ≈ –95 kcal/mol), suggesting their functional coupling. Functional assays in *Escherichia coli* K12 MG1655 revealed that RcsD periplasmic domain represses basal Rcs activity in unstressed cells (~5-fold derepression) yet is essential for stress-induced activation (1.9–2.5-fold). Overexpression of *rcsF* beyond the chromosomal level induces Rcs signal activation only when the RcsD periplasmic domain is present. These findings demonstrate how co-evolution of an auxiliary protein generates novel regulatory function in the Rcs phosphorelay and provide insights into the role of RcsD periplasmic domain in stress signal transduction.

## Introduction

Two-component systems (TCS) are the principal signal transduction machinery in bacteria and have undergone extensive diversification and architectural complexification during evolution [1,2]. Classical TCS consist of a single histidine kinase (HK) and its cognate response regulator (RR), but many lineages — particularly the Enterobacteriaceae — have evolved hybrid and multi-component phosphorelays that incorporate additional sensor domains, auxiliary proteins, and connector modules [3,4].These architectural innovations are thought to arise through gene duplication, domain shuffling, and strong co-evolutionary constraints that maintain signal fidelity within expanded signaling circuits [1,5].

Comparative genomic and phylogenetic studies revealed that interacting components of the same TCS often show correlated evolutionary rates and congruent phylogenies, reflecting functional interdependence and physical interaction [6–8]. Such co-evolution is evident in non-canonical phosphorelays, which often incorporate additional proteins into the classical HK → RR pathway through domain shuffling and fusions [1].

**Fig 1:**
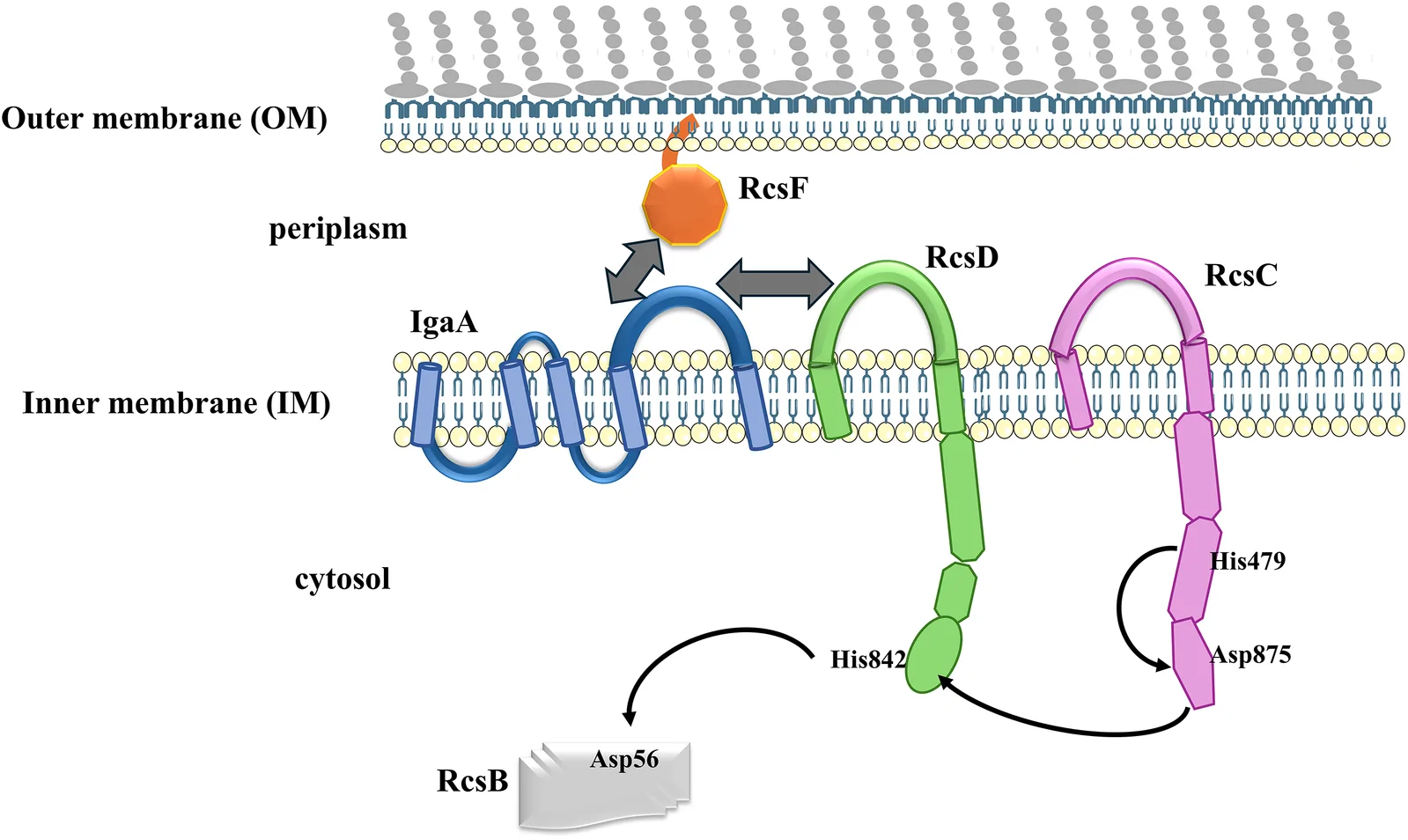
Schematic diagram of the Rcs system in the cell envelope of *E.coli.* Envelope threats are sensed by the OM lipoprotein RcsF, which interacts with the periplamic domain of IgaA located in the IM, causing derepression of the Rcs system. RcsC (HK) autophosphorylates at its conserved histidine residue (His479) and the phopshoryl group is transferred intramolecularly to the aspartate residue (Asp875) then to (His842) of RcsD and finally to Asp56 of RcsB. For simplicity, other interactions of RcsF (with OmpA, BamA), its surface exposed forms and the peptidoglycan (cell wall) residing in the periplasm are not shown. Also, RcsC and RcsD are shown as monomers, although, *in vivo*, they may act as homo or hetero dimers. The direction pf phosphoryl transfer is shown by black arrows. Grey double headed arrows denote protein interactions.

The Regulator of Capsule Synthesis (Rcs) phosphorelay is an important envelope stress response in Enterobacteriaceae, integrating signals from the outer membrane (OM) to modulate capsule synthesis, biofilm formation, motility, and antibiotic resistance (Fig 1) [9,10]. This phosphorelay system involves the sensor kinase RcsC, the phosphotransfer protein RcsD, the response regulator RcsB, and auxiliary regulators RcsF and IgaA [10–14].

RcsF, a 12 kDa OM lipoprotein and the primary stress sensor of the Rcs system, is required for detecting most envelope perturbations, including LPS defects, osmotic stress and β- lactams antibiotics [15,16]. In the presence of Rcs- activating signals, RcsF interacts with the periplasmic domain of IgaA (the inner membrane (IM) negative regulator of the Rcs system) to relieve its repression, thereby derepressing downstream phosphotransfer [14,17,18]. In parallel, RcsF was shown to interact with the essential β- barrel assembly machinery (BamA) and other OM proteins (OmpA, OmpC and OmpF) [19].

The signal detected by RcsF is transmitted to the IM hybrid HK RcsC (which functions also as phosphatase), although the precise mechanism by which the RcsF–IgaA complex communicates with RcsC remains unknown. RcsC autophosphorylates at a conserved histidine residue (His479) in its HK domain in an ATP-dependent manner. The phosphoryl group is then transferred intramolecularly to an aspartate residue (Asp875) within RcsC’s C-terminal receiver (PR) domain. From RcsC ~ P, the phosphoryl is subsequently transferred to a conserved histidine (His842) on the unique C-terminal histidine phosphotransmitter (Hpt) domain of RcsD [10,18,20].

RcsD, is a 100-kDa polytopic IM protein consisting of 890 amino acids. RcsC and RcsD have similar overall architectures, with both containing large periplasmic domains, although RcsC is a hybrid histidine kinase whereas RcsD functions as a His-containing phosphotransfer protein. RcsD features a large periplasmic domain (spanning residues Asn34-Asn308) sandwiched between two transmembrane domains and a large C-terminal cytosolic domain. This cytosolic domain contains a pseudo-histidine-kinase domain, as it lacks a presumed histidine phosphorylation site. It also includes an α/β/loop (ABL) domain, a PAS-like cytoplasmic domain and the essential histidine phosphotransfer (Hpt) domain. After receiving the phosphoryl group from RcsC at His842, RcsD rapidly transfers it to Asp56 of the RR RcsB, thereby activating transcription of the Rcs regulon. The periplasmic and PAS-like domains of RcsD also contribute to signal modulation, likely through direct or indirect interactions with IgaA [10,18]. While the Hpt domain is well-characterized for signal propagation, the periplasmic domain’s role in envelope sensing remains unclear. Recent studies showed that it modulates interactions with IgaA and possibly RcsF [18,21], yet no direct evidence exists for its involvement in stress sensing or signal transduction to downstream components. Whether the periplasmic domain of RcsD plays a role in signal transduction or not represents a key gap in understanding the Rcs phosphorelay architecture and an important evolutionary question for the rationale behind the Rcs system complexity. From an evolutionary perspective, it is not clear why RcsD maintains this large periplasmic domain while its main known role as phosphotransfer occurs in the cytosol, particularly that Rcs system repression predominantly occurs in the cytosol [14] and that IgaA- RcsD periplasmic interaction is not sufficient for Rcs repression [18].

Here, we combine phylogenetic co-evolution analysis, protein docking, molecular dynamics, and domain deletion/ substitution mutagenesis to investigate the role of the RcsD periplasmic domain in signal transduction. We show that RcsF and RcsD co-evolve independently of host speciation and are predicted to form a stable complex. In addition, we show that RcsD periplasmic domain exerts a dual regulatory role of the Rcs system, by negatively regulating basal Rcs activation (in absence of Rcs- inducing cues) and indispensably transducing stress signal across the Rcs system. These findings reveal a new layer of control in the Rcs system with implications for envelope homeostasis and antimicrobial targeting.

## Results

### The evolution of RcsD and RcsF is tightly correlated, independent of species phylogeny

The Rcs system is conserved across the family Enterobacteriaceae [22]. It is characterized by the presence of auxiliary proteins regulating and contributing to the signaling cascades including RcsF, the OM 12 kDa lipoprotein and RcsD, the integral IM phosphotransfer protein. The former has a well characterized role in stress sensing and signal transduction in contrast to the latter. Since both RcsF and RcsD are “extra” components to the HK- RR *duo*, we reasoned that their evolutionary paths might correlate. Accordingly, we investigated whether RcsF (UniProt P69411) and RcsD (UniProt P39838) show evidence of co-evolution, by undergoing a broad homology search across the bacterial kingdom. We searched the complete UniProtKB and UniRef100 [23] databases using both BLASTp [24] and HMMER profile-based searches [25] and also carried out domain-based searches on the ColabFold server [26]. These approaches either returned very few unique hits (≈20 non-redundant sequences) or, conversely, thousands of hits that collapsed into a small number of redundant clusters. After redundancy filtering, the number of genomes encoding both RcsD and RcsF dropped to fewer than ten.

After filtering the outgroup taxa (*Vibrio cholerae, Pasteurella multocida and Pseudomonas aeruginosa*) and species that failed to meet percent identity ≥ 30% and query overage ≥ 70% cutoffs for both RcsD and RcsF proteins, we continued our analysis using manually curated genomes of 32 species from the Enterobacteriaceae family, retrieved from NCBI RefSeq genomes and proteomes.

**Fig 2:**
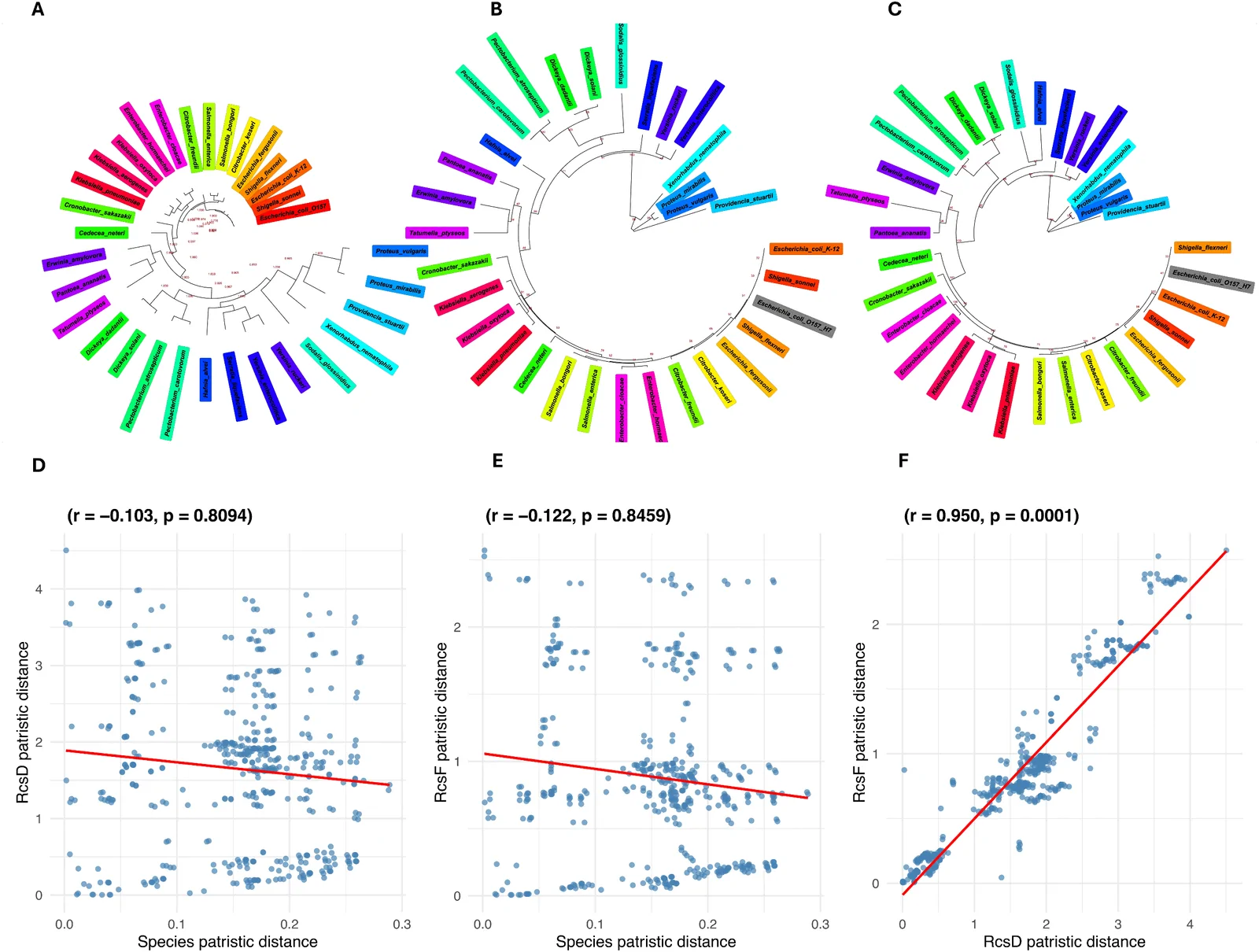
Phylogenetic trees of Enterobacteriaceae species and Rcs proteins. (A) Reference species phylogeny reconstructed from 120 concatenated single-copy marker genes using GTDB-Tk across 32 representative Enterobacteriaceae genomes.(B) Maximum-likelihood phylogeny of RcsD inferred from trimmed multiple sequence alignments using IQ-TREE.(C) Maximum-likelihood phylogeny of RcsF inferred from trimmed multiple sequence alignments using IQ-TREE. Note: Bootstrap support values are shown as proportions (0–1) in (A) and percentages (0–100) in (B) and (C). Together, these trees provide the basis for subsequent correlation analyses (Fig. 2 D-F), enabling comparison of protein-specific evolutionary histories with the underlying species divergence. (D) Correlation between the reference species phylogeny and the RcsD protein phylogeny, shown as a scatterplot of pairwise patristic distances (Mantel test r = −0.103, p < 0.8094). (E) Correlation between the reference species phylogeny and the RcsF protein phylogeny (Mantel test r = −0.122, p < 0.8459). (F) Residual correlation between RcsD and RcsF after statistically controlling for their shared dependence on the species tree (partial Mantel test r = 0.950, p < 0.0001). Each point represents a pairwise species comparison; regression lines are shown for visualization. Together, these results reveal that RcsD and RcsF evolve independently of host speciation but are tightly co-evolving due to functional interdependence.

We constructed a reference species phylogeny from the 32 representative Enterobacteriaceae genomes using GTDB-Tk (Fig 2A). Maximum-likelihood phylogenies were then inferred for RcsD and RcsF from trimmed multiple sequence alignments (Figs 2B and 2C). To evaluate phylogenetic congruence, patristic distance matrices were compared using Mantel and partial Mantel tests (Pearson correlation, 9,999 permutations). Surprisingly, neither RcsD nor RcsF phylogenies showed significant congruence with the species tree (Mantel test: r = −0.103, p = 0.821; r = −0.122, p = 0.840, respectively), indicating that their evolutionary trajectories are not constrained by host speciation. However, a partial Mantel test revealed an exceptionally strong co-evolutionary relationship between RcsD and RcsF (partial r = 0.950, *p* < 0.0001), even after controlling shared phylogenetic history. This suggests that the two proteins are under coordinated selective pressure, likely due to their contribution to a specific function within the Rcs phosphorelay system. In contrast, RcsC (the IM HK) and RcsF showed strong phylogenetic congruence with each other (Mantel r = 0.958, p = 0.0001), but both proteins were also strongly congruent with the GTDB species tree (RcsC vs species tree: r = 0.895, p = 0.0001; RcsF vs species tree: r = 0.902, p = 0.0001). After controlling for species phylogeny, the RcsC–RcsF association was reduced slightly but remained significant (partial Mantel r = 0.785, p = 0.0001; S1 Fig). Since the RcsD–RcsF relation remained exceptionally strong despite weak or non-significant congruence with the species tree, we hypothesized that a more specific species-independent evolutionary association exists between RcsD and RcsF (rather than a general correlation among conserved Rcs components). These results suggest direct evidence for their co-evolutionary relationship (visual representations of the correlation analyses are shown in Fig 2 (panels D–F).

### RcsD periplasmic domain and RcsF are predicted to exhibit high mutual affinity

Building on the co-evolutionary evidence suggesting a functional interdependence between RcsD and RcsF, we hypothesized that RcsD periplasmic domain (Asn34- Asn308), could contribute to signal sensing and/ or transduction, mediated through mutual affinity with RcsF. Accordingly, we screened the primary sequence of *Escherichia coli* K-12 substr. MG1655 RcsF and RcsD to determine which residues are likely to contribute to a plausible physical interaction between them. After an initial screening using both Scannet and AWSEM-MD, we identified residues showing a score greater than 0.5 in both methods and classified the residues into four groups based on their spatial proximity in the 3D structure. The spatial distribution of these high-scoring residues (score > 0.5) within the periplasmic domain of RcsD is shown in S2 Fig., where residues are grouped based on their proximity and localization on the same or different surface regions of the protein.

We conducted four runs of docking, each with different residues. In the first run, we chose Pro222, Gly223, Asp253, Ala254, and Lys171 in the RcsD periplasmic domain and docked them against all residues of RcsF. The second run involved the same residues from RcsD (Pro222, Gly223, Asp253, Ala254, and Lys171) docked against residues Pro34 through Ala46 of RcsF. In the third run, we picked residues Asp48, Tyr51, and Asn140 of RcsD and docked them against all residues of RcsF. Finally, in the fourth run, residues Asp48, Tyr51, and Asn140 of RcsD were docked against residues Pro34 through Ala46 of RcsF. We conducted docking for each of the four scenarios mentioned above using the HADDOCK 2.4 web server. After docking, we analyzed the complexes formed and selected the best ones based on their Z-scores and ΔG scores (where lower scores indicate better binding). The best RcsD- RcsF periplasmic complex involved the following residues: Pro222, Gly223, Asp253, Ala254, and Lys171 in the periplasmic domain of RcsD, with all residues of RcsF. To compare the predicted RcsD- RcsF interaction with other interactions, we performed docking between the IgaA periplasmic domain (Pro359-Arg654) and RcsF, since this interaction was proved experimentally [14,17,21]. As a benchmark for the docking pipeline, the HADDOCK-modeled RcsF–IgaA complex was compared with the experimentally determined RcsF–IgaA periplasmic complex (PDB: 9BIY). Superposition based on 259 Cα atoms of IgaA yielded an RMSD of 0.349 Å. Using a 5 Å interface cutoff, the model partially recovered the experimental IgaA interaction surface, with 13 of 21 interface residues shared between the modeled and experimental complexes (despite RcsF showing a different conformation) (S3 Fig.). Additionally, we conducted docking experiments between RcsF and the MalF periplasmic domain (Asn93-Lys275) for which no interactions were reported previously and are unlikely to occur. The corresponding ΔG Scores are mentioned in Table 1, indicating a negative score (higher stability) for RcsF- RcsD periplasmic complex and RcsF- IgaA complex (in contrast to RcsF- MalFperiplasmic complex). This result suggests that the periplasmic domain of RcsD and RcsF are functionally correlated and might interact directly oy indirectly with RcsF *in vivo* [21].

**Table 1:** ΔG Scores of Docking Experiments Between RcsF and RcsD periplasmic.

Complex	ΔG	
RcsF-MalF-periplasmic complex	49.886	Negative control
RcsF-RcsD periplasmic complex	−10.552	Test
RcsF- IgaA periplasmic complex	−5.241	Positive control

### The predicted RcsD-RcsF interaction remains stable throughout molecular dynamics simulations

**Fig 3:**
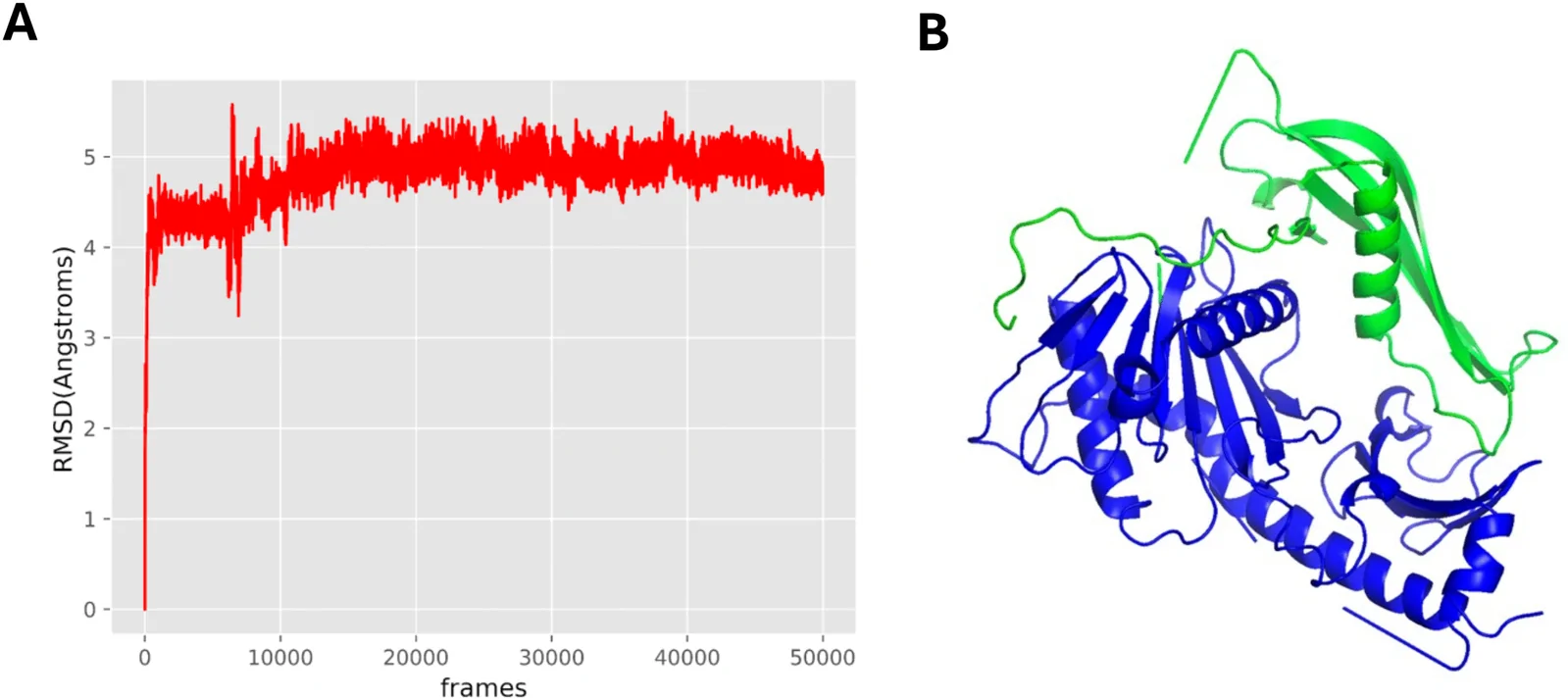
Predicted RcsF- RcsD interaction (A) The computed root mean squared deviation (RMSD) of protein backbone Cα over the course of 500 nanoseconds. (B) Putative RcsF-RcsD periplasmic complex (RcsF: green, RcsD periplasmic: Blue).

Based on the docking results, we evaluated the stability of the RcsF- RcsD complex showing the best Z-score and ΔG, by measuring the root mean squared deviation (RMSD) of its backbone alpha carbons (Cα) throughout molecular dynamics (MD) simulation. Our analysis revealed that the protein reached an equilibrium conformation within 100 nanoseconds, with a notable deviation of 5 angstroms from the initial frame, as shown in Fig 3A.

To confirm the stability of the RcsF- RcsD periplasmic interaction (Fig 3B) as suggested by the RMSD analysis, we calculated the binding energy of the complex. The binding energy analysis using the MM-GBSA method revealed a strong binding affinity between RcsD and RcsF proteins, with an energy value of −95.43 kcal/mol.

The stability of the RcsF- RcsD binding is primarily driven by dominant gas-phase electrostatic attractions ΔE_ele_ = −1314.04 kcal/mol, which significantly outweigh van der Waals contributions. While the complex faces a substantial desolvation penalty ΔE_GB_ = 1377.29 kcal/mol typical of highly polar interfaces, the total binding free energy remains strongly favorable at ΔG_Total_ −95.34 kcal/mol (Table 2).

**Table 2:** Calculated binding free energy of the predicted interaction between RcsD-RcsF using MM-GBSA method.

Proteins	ΔE_vdW_	ΔE_ele_	ΔE_GB_	ΔE_Surf_	ΔG_Gas_	ΔG_Solv_	ΔG_Total_
RcsD-RcsF	−137.57 ± 9.4	−1314.04 ± 58.1	1377.29 ± 51.89	−21.01 ± 0.89	−1451.61 ± 58.48	1356.27 ± 51.49	−95.34 ± 10.26

Both RMSD and binding energy analyses suggest that the RcsD- RcsF interaction is stable with a strong binding affinity, proposing their possible functional association.

### RcsD periplasmic domain plays an inhibitory role in basal Rcs signalling

**Fig 4:**
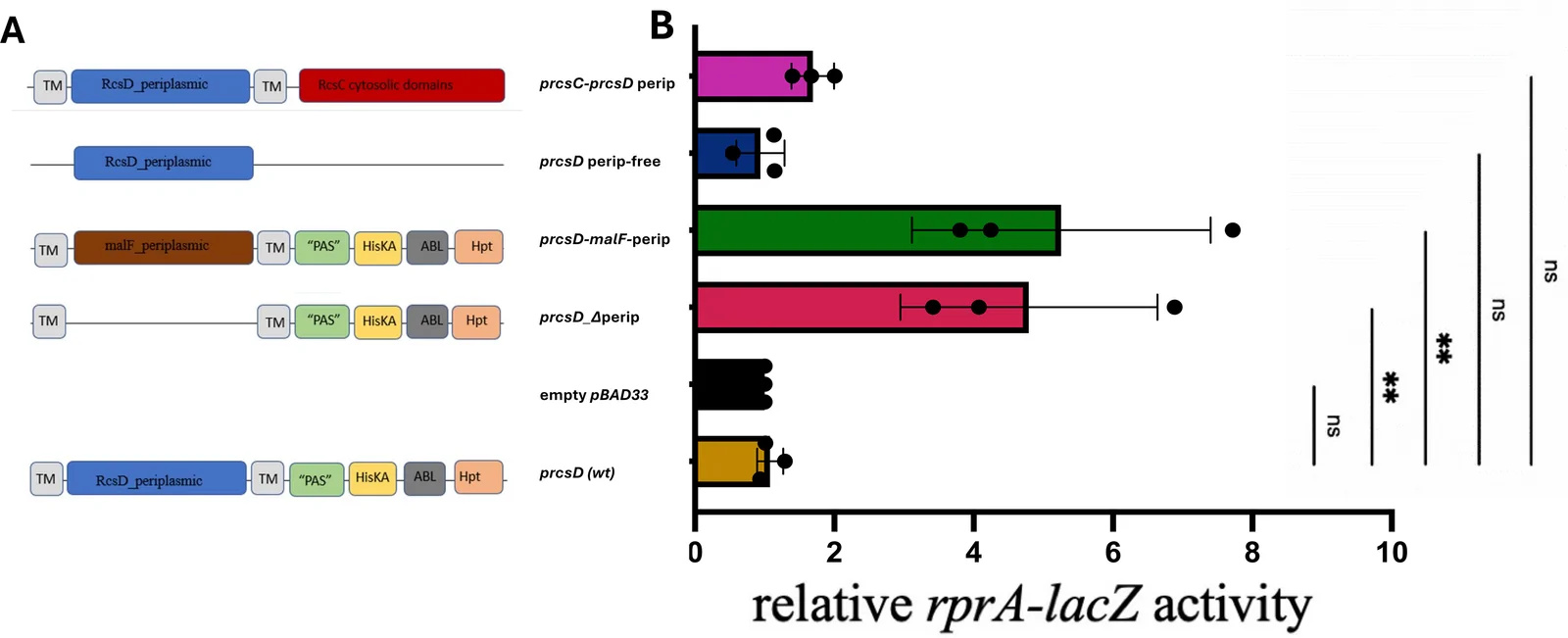
Effect of RcsD periplasmic domain on basal Rcs signaling. (A) RcsD variants generated during this study to assess the role of RcsD periplasmic domain. (B) β- galactosidase assay (relative *rprA*-*lacZ* activity) indicates the activation of Rcs system (normalized to the basal activation of the isogenic strain transformed with empty pBAD33). Satistical comparaison were done using ANOVA analysis and *post hoc* tests. Double asterisks (**) indicate *p* ≤ 0.01 while (ns) denote non- statistically significant.

Our phylogenetic co-evolution analysis and computational predictions from docking and MD simulations suggested a possible genetic and/ or functional correlation of RcsF and RcsD. According to the localization of RcsF and RcsD in their respective subcellular compartments (Fig 1), contribution of RcsF– RcsD in a particular signalling or signal regulation role must physically occur in the periplasmic space (the space confined between the OM and IM), implicating the RcsD periplasmic domain as a key player. We therefore hypothesized that RcsF and RcsD together modulates basal Rcs signaling *in vivo*. To test this experimentally, we examined the impact of deleting the RcsD periplasmic domain (Asn43–Asn308) (p*rcsD*- Δperip) (Fig 4A) on Rcs system activity using a β-galactosidase reporter assay in *E. coli* DH300 [27]. *E.coli* DH300 carries the *rprA* promoter-*lacZ* fusion at the lambda attachment site and is widely used to monitor Rcs system activation as the small regulatory RNA RprA is regulated by RcsB [14,17,27]. We observed 5-fold activation of the Rcs system (Fig 4B), corroborating previous findings [18]. To confirm this result and rule out the possibility that the observed Rcs activation upon deletion of the RcsD periplasmic domain results from the proximity of the two transmembrane domains, we substituted the RcsD periplasmic domain with the MalF periplasmic domain (p*rcsD*-*malF* perip). MalF periplasmic domain has no reported interaction with RcsF, is of comparable molecular mass to RcsD periplasmic domain and thus can mimic the spatial spacing of the RcsD periplasmic domain without interacting with RcsF. Again, we observed 5-fold activation of the Rcs system, similar to what we observe when the RcsD periplasmic domain was deleted (Fig 4B). These results suggested that RcsD periplasmic domain might negatively regulate the signal flow to the cytosolic components of the Rcs system. To confirm further the role of RcsD periplasmic domain, independently of its transmembrane and cytosolic domains, we generated a free RcsD periplasmic domain (p*rcsD* perip-free) (exported to the periplasm through an N-terminal export signal) and RcsD periplasmic domain in RcsC backbone (p*rcsC*- *rcsD*perip). Interestingly, both didn’t cause any significant activation compared to the wild type RcsD as shown in Fig 4.

The increased basal activity of the Rcs system upon substitution or deletion of the RcsD periplasmic domain and its absence in other variants, confirm the inhibitory effect of the RcsD periplasmic domain. These findings support the previous work by [18] and implies that, the minimal RcsF- IgaA interaction responsible of the basal Rcs activity might be controlled by this domain.

To confirm that the observed differences in Rcs activation were not due to variations in protein expression, we compared the protein expression level of the abovementioned *rcsD* mutants after induction from their corresponding plasmids. Expression levels of all RcsD variants were comparable to the wild-type RcsD expressed from the same plasmid, with the exception of the free periplasmic domain which could not be definitively quantified (S4 and S5 Fig). Overall, our results indicate the observed differences in Rcs activation are due to RcsD periplasmic domain function, rather than the differences in protein expression levels.

### RcsD periplasmic domain enhances stress-induced signaling

**Fig 5:**
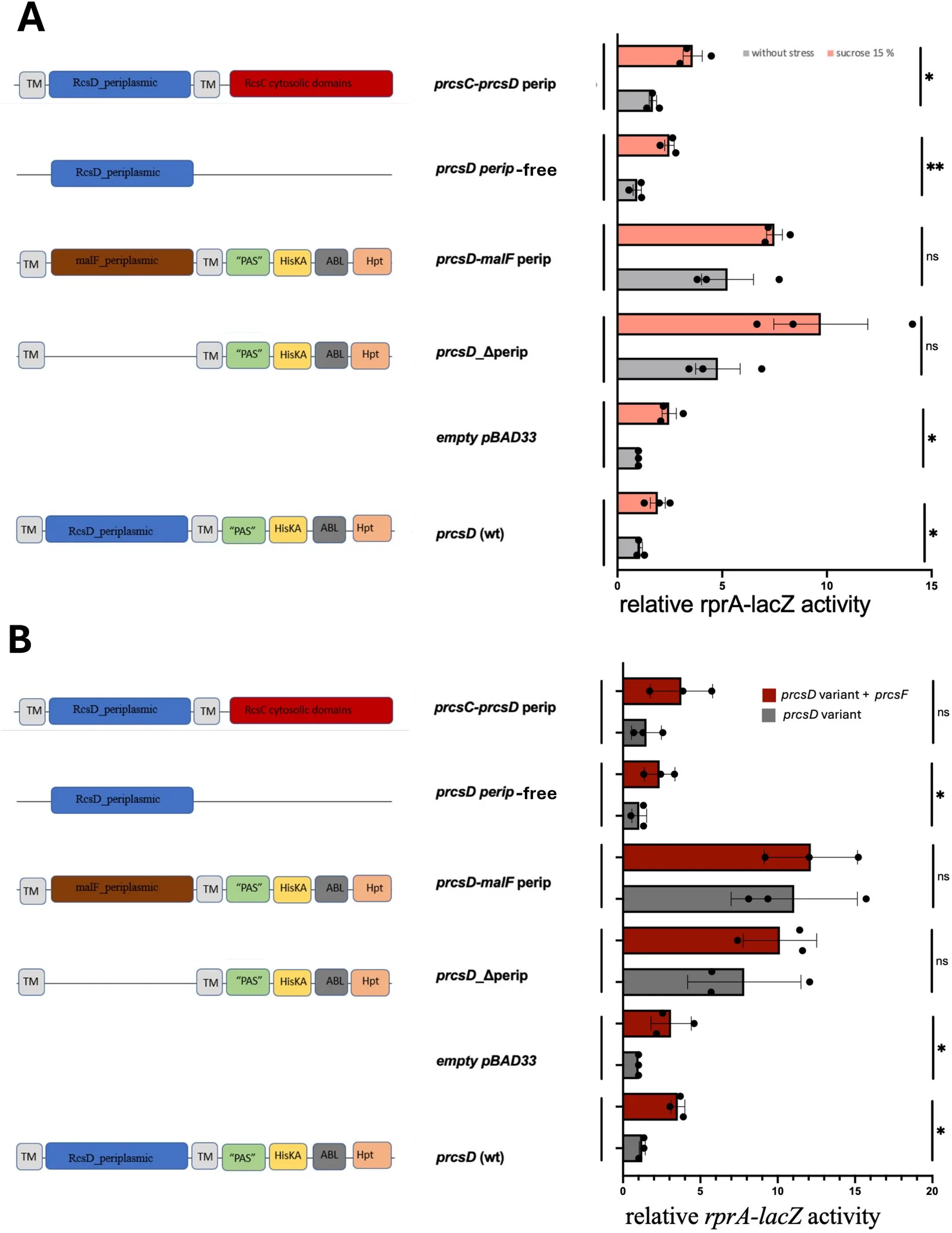
Effect of RcsD periplasmic domain on Rcs response to stress. β- galactosidase assay (relative *rprA-lacZ* activity) indicates the relative activation of Rcs (normalized to the basal activation of the isogenic strain transformed with empty pBAD33). (A) when *rcsF* is expressed from the bacterial chromosome, (B) upon co-expression of p*rcsF*. Statistical significance for the indicated pairwise comparisons was determined using two-sample t-tests. Single asterisks (*) indicate *p*-values ≤ 0.05, double asterisks (**) *p* ≤ 0.01, and (ns) non- statistically significant.

Next, we wondered if RcsD periplasmic domain alters Rcs activation in response to stress. We reasoned that, if RcsD could receive a signal sensed by RcsF, RcsD periplasmic domain could be essential for Rcs system activation. Accordingly, we repeated the previous experiment by adding 15% sucrose, which has been reported to induce an RcsF- sensed osmotic shock in *E. coli* and activate the Rcs system [28]. The results revealed that both the substitution and deletion of the RcsD periplasmic domain (p*rcsD-malF* perip and p*rcsD*-Δperip) did not lead to an increased response in Rcs activation after osmotic shock. In contrast, wild type RcsD showed a 1.9-fold increase in Rcs activity in the presence of sucrose compared to the no-stress condition. On the other hand, expressing the RcsD periplasmic domain either freely or within the RcsC backbone (p*rcsD*-perip-free and *prcsC-rcsDperip*) resulted in an increased response to stress, with 2.5-fold and 2.1-fold increase, respectively (Fig 5A). The increased Rcs response to stress in strains transformed with p*rscC*- *rscD*perip and p*rcsD*-perip-free and its absence in those transformed with p*rcsD-malF* perip and p*rcsD*-Dperip suggests a role of the periplasmic domain of RcsD in response to RcsF- sensed activation signals.

In other words, when present alone or anchored to RcsC or RcsD backbones, RcsD periplasmic domain enhances response to stress in contrast to RcsD transmembrane and cytosolic domains where the periplasmic domain was deleted or substituted (RcsD perip and RcsD-MalF). This result suggests that the RcsD periplasmic domain may be required for activation of the Rcs system in response to specific cues.

### RcsF-dependent activation of the Rcs system requires the RcsD periplasmic domain

Taken together, our findings support a possible signal transmission from RcsF to RcsD, as evidenced by: 1- The phylogenetic analyses showing that RcsD and RcsF exhibit significant co-evolution beyond shared speciation events. 2- The predicted high affinity association between RcsF and RcsD using protein-protein docking and molecular dynamics, 3- The experimental results supporting a role of RcsD periplasmic domain in Rcs response to stress. Because RcsF is the stress sensor of the Rcs system, we can anticipate that the effect of RcsD periplasmic domain on stress response could be mediated by the interaction (direct or indirect) of RcsF (expressed from the chromosome) with RcsD periplasmic domain (expressed from either p*rcsD* wild type or p*rcsD*-perip -free or p*rcsC*-Dperip). This predicted interaction could be a part of the reported RcsF-IgaA- RcsD complex [21].

To confirm our findings, we wondered if providing the Rcs system with extra copies of *rcsF* (through expression from a medium copy number plasmid) would lead to an enhanced Rcs system activation. To this end, we co-transformed the medium copy number plasmid pAM238 (*rcsF*-pSC202) [17] with each of the RcsD variants described above and assessed Rcs system activity.

As shown in Fig 5B, in the presence of p*rcsF*, we did not observe a significant increase in Rcs system activation upon co-expression of either *rcsD* – *malF*perip or *rcsD*- Δperip in contrast to co-expression of wild type *rcsD* with *rcsF* (2.5 fold increase). On the other hand, free RcsD periplasmic domain (expressed from p*rcsD*-perip) with RcsF showed a 2.4-fold activation, similar to the wild-type activation level. This supports the hypothesis that the RcsD periplasmic domain plays a role in signal transfer. Finally, the RcsD periplasmic domain within the RcsC backbone (expressed from p*rcsC*-Dperip) did not show a significant increase in system activation in presence of extra copies of RcsF (possible explanation in the discussion section).

Together, these results strongly suggest that the RcsD periplasmic domain plays a role in stress-mediated activation of the Rcs system. We can infer that, RcsD periplasmic domain is indispensable for stress signal transduction and Rcs activation in presence of RcsF.

## Discussion

Phosphorelay system are one of the main factors conferring to bacteria their surprising adaptability. Evolution of different phosphorelay system protein components enables their robustness and remarkable specificity while minimizing unspecific cross- talks providing several points for regulation and control.

The Rcs system is one of three envelope stress response phosphorelay system conserved in the family Enterobacteriaceae [22]. It constitutes one of the few phosphorelay **system** where the transfer of the phosphoryl group is mediated by an intermediate protein (RcsD) between the HK and the RR, while most signals are sensed by an outer membrane lipoprotein, RcsF [10].

In this study, we investigated the evolution of RcsD and RcsF within the family Enterobacteriaceae. A previous study investigating the evolution of IgaA (the essential IM negative regulator of the Rcs system) in some members of the *Enterobacterales* order suggested that, from an evolutionary viewpoint, RcsF and IgaA might have been integrated later in the Rcs system [8]. In parallel, previous research showed the interaction between IgaA-RcsF [14,17] and IgaA-RcsD [18]. This led us to ask the question if, in turn, RcsF and RcsD are evolutionary related and play a coordinated role in stress sensing and response in the Rcs system.

Our phylogenetic analysis revealed a strong co-evolutionary coupling between RcsF and RcsD across 32 Enterobacteriaceae genomes, independent of host speciation (partial Mantel r = 0.950, p < 0.0001). Neither the RcsD nor RcsF phylogenies showed significant congruence with the species tree (Mantel r = −0.103, *p* = 0.821 and r = −0.122, *p* = 0.840, respectively), suggesting that their evolutionary trajectories are driven by functional interdependence rather than shared phylogenetic history (in contrast to RcsC and RcsF pair). This tight co-evolution suggests either selective pressure to maintain coordinated interactions within the Rcs phosphorelay or a functional coupling, between RcsF and RcsD. In other words, our results could lead to the hypothesis of a plausible RcsF- RcsD physical interaction (that could occur, alone or in complex with other proteins including IgaA) or a functional correlation (where RcsD and RcsF do not interact directly but work in concert for signal sensing, regulation and/ or transmission).

The currently accepted model explaining Rcs system activation, is the sensing of inducing cues by RcsF, triggering its interaction with the large periplasmic domain of IgaA [14,17]. This interaction leads to de-repression of the system, firing of the signalling cascade by autophosphorylation of RcsC, transferring the phosphoryl group to RcsD and finally to RcsB, the RR. This model suggests RcsF- IgaA interaction as the main transmitter of the signal from the cell envelope to the cytosol and the main trigger for Rcs activation. In addition, previous work [18] suggested that the interaction of IgaA and RcsD in the periplasm is an indispensable anchor for full IgaA- mediated repression. Another study reported the co-purification of RcsF and RcsD only when a full length IgaA is present [21]. Taken together with our evolutionary analysis, these results suggest a functional correlation between RcsD and RcsF, mediated through IgaA periplasmic domain as the main anchor point for repression and de-repression.

Building on this evolutionary evidence, our docking analysis suggests a hypothesis that RcsF and RcsD form stable complexes. Using HADDOCK 2.4, we found that the complex involving Pro222, Gly223, Asp253, Ala254, and Lys171 of RcsD docked against all residues of RcsF, had the most favorable binding characteristics with a ΔG score of −10.552 kcal/mol. This predicted interaction score was comparable to the one predicted for an experimentally proven complex (RcsF-IgaA complex) in contrast to a hypothetical (RcsF-MalF complex), with the latter showing significantly less favorable binding energy. To assess dynamic stability, 500-ns MD simulations showed equilibration within 100 ns (RMSD deviation ~5 Å), with MM-GBSA binding energy of −95.34 kcal/mol, driven mainly by electrostatic interactions (ΔEele = −1314.04 kcal/mol) despite unfavorable solvation. Nevertheless, we are cautious to interpret these results as a direct complex formation between RcsF and RcsD independent of the reported RcsF-IgaA or RcsD-IgaA or RcsF-IgaA-RcsD complexes [18,21]. Instead, we hypothesize that the predicted affinity between RcsF and RcsD periplasmic domain suggests their functional coupling to exert a regulatory or signal transducing role within the signaling cascade.

The above results motivated us to explore if the periplasmic domain of RcsD contributes to signal sensing and/ or transmission from RcsF in the model bacterium *E.coli* K12 MG1655. Our mutational analysis showed that, in the absence of Rcs inducing cues, the RcsD periplasmic domain exerts an inhibitory effect on Rcs system basal activity. Absence of the RcsD periplasmic domain led to a significant increase in Rcs system activity, consistent with previous findings reporting that a periplasmic IgaA- RcsD interaction serves as an anchor for Rcs system repression (albeit not sufficient on its own to fully repress the system) [18]. The substitution of the RcsD periplasmic domain with the MalF periplasmic domain mimicked the deletion effect, further corroborating the regulatory function of the RcsD periplasmic domain under non- stress conditions and out ruling that the increased basal activity is caused by the physical proximity of RcsD transmembrane domains. Both wild-type RcsD and all RcsD variants (except the free RcsD periplasmic domain), showed comparable protein expression levels as determined by western blot (S4 and S5 Fig.).

When subjected to sucrose osmotic stress, strains harboring mutants with deleted or substituted RcsD periplasmic domain showed only a mild but statistically non-significant Rcs system activation. On the other hand, the RcsD periplasmic domain either in its soluble form or within the RcsC backbone resulted in a statistically significant increase of Rcs system activity, similar to wild type RcsD. Collectively, these results suggest that the periplasmic domain of RcsD, may have an additional role in stress signaling, beyond its role in assisting IgaA- mediated repression of the Rcs system in the absence of inducing cues. A recent study showed that some signals might activate the Rcs system independently of RcsF, by weakening RcsD- IgaA interaction [29]. The role of RcsD periplasmic domain in this case should be studied further.

Presence of RcsD with extra copies of RcsF showed 2.5-fold activation when both wild types RcsD and RcsF are overexpressed or when the free RcsD periplasmic domain is overexpressed with RcsF. On the other hand, the absence of the RcsD periplasmic domain, either by deletion or substitution, with extracopies of RcsF did not result in a significant increase in Rcs system activity, suggesting a model where RcsD periplasmic domain is required for appropriate stress response, probably by partially receiving the stress signal from RcsF. RcsD might receive the stress signal from RcsF-IgaA complex or from RcsF alone. Whether the role of RcsD periplasmic domain is to receive the stress signal from RcsF and transduce it to RcsC or facilitate RcsF interaction with IgaA or has an independent role remains to be investigated. These findings can be viewed in the light of the RcsF- IgaA and IgaA- RcsD model suggested by other groups [18,21]. as a dual regulatory role of RcsD periplasmic domain where it contributes to the full Rcs system repression under non- stress conditions (through its complex with IgaA) while it becomes involved (directly or indirectly) in signal transmission from RcsF or from RcsF- IgaA complex.

However, our suggested role of the RcsD periplasmic domain in concert with RcsF contrasts with the results of Sato et al. (2017), who reported that the RcsD periplasmic domain, exported through fusion to maltose-binding protein, failed to titrate the activating effect of a constitutively active RcsF mutant [30]. In our experience and as outlined by the same authors, free RcsD periplasmic domain exhibits low stability at the protein level (S4 and S5 Fig.), and thus the absence of its effect could not be assessed with the high activation caused by the constitutively active RcsF mutant used by Sato and co- workers.

Surprisingly, RcsD periplasmic domain anchored to RcsC backbone when expressed with extracopies of RcsF did not lead to Rcs system activation, suggesting that RcsD transmembrane domains and/ or cytosolic domain are required for RcsD – mediated stress response, possibly independent of the known phosphotransfer role. A second possibility would be that, in the presence of activating signals, the RcsD periplasmic domain induces conformational changes to the RcsC backbone, resulting in increase of RcsC phosphatase activity thus preventing or neutralizing Rcs system activation.

Another possible explanation of the observed increase in Rcs activity mediated by overexpression of *rcsD* or *rcsD* mutants could be IgaA titration as suggested by [18]. In other words, the extracopies provided from ectopic expression of *rcsD* or *rcsD* periplasmic domain could interact with IgaA, thus “pulling it” out of its downstream partners and de-repressing the Rcs system. However, we believe that the IgaA- titrating effect could only partially explain our results. From one side, a model where RcsD or RcsD variants titrate IgaA will lead to Rcs activation at rest (in the absence of stress) (which is not the case as shown in Fig. 4A). On the other hand, titration of IgaA should lead to a higher fold activation of the Rcs system that the one observed here as the Rcs system is particularly sensitive to IgaA- mediated negative regulation. Even if true, if we take in consideration the copy number per cell of RcsD (179 molecules/cell), RcsF (1180 molecules/cell) and IgaA (78 molecules/cell) [31], extra RcsD (expressed from the plasmid) will associate with all endogenous copies of IgaA leading to freeing up RcsD expressed from the chromosomal copy, that could then be responsible of the activating effect. Again, this activating effect, only observed under stress conditions, suggests a role in transducing stress signal rather than just disrupting the basal RcsF-IgaA-RcsD regulatory axis.

In conclusion, our work suggests a dual role for the RcsD periplasmic domain depending on the presence or absence of Rcs- activating stress signals. In the absence of stress signals, it assists IgaA in inhibiting Rcs system activation (together with its cytosolic domain), thereby maintaining it at its minimal activation level. Conversely, in the presence of activating signals, RcsD periplasmic domain receives the stress signals (directly or indirectly) from RcsF and/or RcsF- IgaA complex facilitating signal transfer. Whether RcsF and RcsD form a binary complex or a ternary complex with IgaA and the nature/ activity of this complex remain to be investigated. In addition, a detailed mapping of RcsD periplasmic domain residues contributing to signal transduction would add an extra layer of evidence to the data reported here by mutating specific residues leading to signal alteration of the function of RcsF- RcsD axis.

## Materials and methods

### Phylogenetic tree construction and mantel test analysis of RcsF and RcsD

To investigate whether RcsF and RcsD show evidence of co-evolution, we attempted a broad homology search across the bacterial kingdom. We searched the complete UniProtKB and UniRef100 [32] databases using both BLASTp [24] and HMMER profile-based searches [25], and also carried out domain-based searches on the ColabFold server [26].

Due to the very small number of non- redundant hits obtained, we curated a manual representative set to allow meaningful phylogenetic comparison. We chose 39 species spanning the Enterobacteriaceae, and with three additional outgroup taxa (*Vibrio cholerae, Pasteurella multocida* and *Pseudomonas aeruginosa)*, giving a total of 42 genomes (S1 Table). These were retrieved from NCBI RefSeq [33](genomes and proteomes).

Reference sequences for RcsD (UniProt P39838) and RcsF (UniProt P69411) were obtained from UniProt [23] and used as queries for BLASTp [24] searches against the selected genomes. Hits were filtered to retain sequences with at least 25% identity.

For the species phylogeny, we used GTDB-Tk [34] (120 conserved bacterial markers) implemented on the KBase [35] platform, producing a robust reference tree. Protein homologs for RcsD and RcsF were extracted from the curated genomes, aligned with MAFFT [36], and trimmed with trimAl [37] to remove poorly aligned positions. IQ-TREE v2.4.0 [38] was used to infer maximum-likelihood trees for each protein under automatic model selection.

Finally, to assess co-evolutionary relationships, we calculated patristic distance matrices for the species tree, RcsD tree, and RcsF tree, and compared them using the Mantel test and partial Mantel test (Pearson correlation, 9,999 permutations), as implemented in the R packages ape [39] and vegan [40]. These tests allowed us to evaluate whether the evolutionary trajectories of RcsD and RcsF remain correlated after controlling for the shared species phylogeny.

To test whether the observed RcsD–RcsF correlation was specific or simply reflected congruence among conserved Rcs pathway components, the same phylogenetic and Mantel/partial Mantel workflow was also applied to RcsC and RcsF. RcsC homologs were identified from the same curated genome set using the *E. coli* RcsC reference sequence, aligned, trimmed, and analyzed using the same tree-building and distance-correlation procedures described above.

### Prediction of RcsD- RcsF interaction using docking and molecular dynamics

To perform docking between the periplasmic domain of RcsD (Asn43- Asn308) (UniProt ID: P39838, AlphaFold ID: AF-P39838-F1) and RcsF (UniProt ID: P69411, PDBe ID: 2l8y), we conducted two analyses to predict the possible interacting residues. They were then used as active site residues in protein-protein docking. First, we employed ScanNet [41] a machine-learning model that predicts which residues are likely to be involved in interactions based on several parameters, including solvent accessibility and charge status. Second, we utilized AWSEM-MD [42], a model based on local frustration analysis. Following that, we performed docking analysis on the selected residues from the previous step using HADDOCK 2.4 [43,44] which generated several structures in different poses with their corresponding z-scores. As a positive control, we performed docking between IgaA (UniProt ID: P45800, AlphaFold ID: AF-P45800-F1) periplasmic domain (Pro359- Arg654) and RcsF, which were shown to interact experimentally [14]. Additionally, as a negative control, we conducted docking experiments between RcsF and the MalF (UniProt ID: P02916, PDBe ID: 2R6G) periplasmic domain, for which no interactions were reported previously and are unlikely to interact.

We chose two potential RcsD-RcsF complex structures with the lowest z-scores and ΔG values and subjected them to 500 nanoseconds of molecular dynamics (MD) simulations using OpenMM [45]. The complexes were solvated in a cubic box with a 10Å buffer distance from the protein surface, utilizing the TIP3P water model. To neutralize the system charge, sufficient counter ions were added. The protein topology was generated using the Amber 14SB force field. The solvated system underwent energy minimization to eliminate any steric clashes. Subsequently, the system was heated to 300 K over 0.5 nanoseconds in the *NVT* ensemble. Following this, the system was equilibrated in the *NPT* ensemble for 1.0 nanoseconds, employing the MonteCarloBarostat for pressure control and the Langevin Integrator for temperature regulation. The equilibrated system then proceeded to a 500-nanosecond MD simulation. The processing and analysis of the resulting MD trajectories were conducted using Pytraj [46]. Protein conformation stability was assessed by calculating the root mean squared deviation (RMSD) relative to the initial frame of the MD trajectory.

The binding free energy analysis of the RcsD-RcsF complex system on the last 5 (250 frames) nanoseconds was performed by the MMGBSA from AmberTools. The binding energy was calculated using the following formula: ΔG(bind) =G(RL) – G(Ra) – G(Rb). The ΔGbind is further decomposed into enthalpy and entropy terms as following: ΔG(bind) =ΔH – TΔS. The Enthalpic term is further decomposed into molecular mechanics energy term and solvation free energy as following: ΔG(bind)= ΔE(MM) + ΔG(Sol) – TΔS. The molecular mechanics energy term and the solvation free energy are further decomposed into: ΔE(MM) = ΔE(int) + ΔE(ele) + ΔE(vdw). The solvation free energy is solved either by linearised Poisson Boltzmann or Generalized Born equation along with an empirical term added to the equation to calculate the hydrophobic contribution ΔG(SA) between the solute and solvent model:


$$\begin{array}{c} \Delta \text{G}(\text{sol})\;=\;\Delta \text{G}(\text{PB}/\text{GB})\;+\;\Delta \text{G}(\text{SA}) \end{array}$$

$$\begin{array}{c} \Delta \text{G}(\text{SA})\;=\;\gamma \;.\text{ SASA }+\text{ b} \end{array}$$

### Design and generation of RcsD periplasmic domain mutants

To gain insights into the role of RcsD periplasmic domain in Rcs system activation and stress response, six variants of RcsD were designed. The nucleotide sequence of the designed *rcsD* variants were synthesized by (Gene Universal Inc, China) and supplied cloned in pUC57. The *rcsD* variants were amplified using Phusion high fidelity polymerase (Thermo) following the standard protocol with few modifications and cloned between SacI and XbaI restriction sites in the pNH431, as previously described (Hussein et al., 2018). This plasmid is derived from the chloramphenicol-resistant, L- arabinose inducible pBAD33 plasmid backbone (Guzman et al., 1995) and was modified by deleting the original XbaI site and adding at the C-terminus a 5x-histidine tag flanked by XbaI and KpnI restriction sites. Accordingly, inserts cloned between SacI and XbaI sites will be expressed as fusion proteins with a C-terminal 6 × His tag according to the manufacturer’s instructions. All constructs were verified by sequencing using the Sanger sequencing facility (Macrogen, Korea). Plasmids used and generated in this study are listed in (S2 Table).

### Generation of strains and culture conditions

To monitor the activation of the Rcs system, the recombinant plasmids purified from the previous steps were transformed into chemically competent *E. coli* MG1655 DH300 [27] which carries the *rprA*::*lacZ* fusion at the lambda attachment site and was provided as a gift from the Collet laboratory (Université catholique de Louvain, Belgium) to Nahla Hussein. The *rprA* promoter is regulated by the Rcs system response regulator RcsB (Fig 1). Accordingly, its activation results in increased β- galactosidase activity that can be monitored using the assay described below. The list of strains used and generated in this study is described in (S3 Table).

Unless otherwise specified, all strains were cultured in Luria Bertani (LB) -Miller medium or on LB-Miller agar (Sigma-Aldrich) at 37°C. Antibiotics were added when necessary: as chloramphenicol at a concentration of 25 μg/μl and spectinomycin at 100 μg/ml. If combining two antibiotics, the concentrations were halved accordingly. Isopropyl ß-D-1-thiogalactopyranoside (IPTG) and L-arabinose were added as inducers at concentrations of 100 μM and 0.2%, respectively. For all experiments, we cultured the corresponding strains overnight in the presence of either antibiotics and/ or inducers. Subsequently, the overnight cultures were diluted 1/100 in fresh LB-Miller medium supplemented with the corresponding antibiotic and/ or inducers. The growth was assessed by measuring the optical density at 600 nm. Whenever needed, the strains were treated at OD600 of 0.4–0.5 with 15% sucrose for 1 hour as Rcs-inducing cues.

### Assessment of Rcs system activation by β-galactosidase assay

Activation of the Rcs system was assessed by measuring of β-Galactosidase activity of the *rprA*::*lacZ* fusion according to [48] with some modifications. AO strains, referring to the *E. coli* K12 MG1655 DH300-derived strains generated in this study and listed in S3 Table, were grown in the presence of the appropriate inducer (100 μM IPTG) and/ or 0.2% L-arabinose) and/ or antibiotics (chloramphenicol and/ or spectinomycin) until the mid-log phase. Then, 20 μl of each sample were permeabilized for at least one hour by incubation with 80 μl of permeabilization solution (100 mM Na_2_HPO_4_, 20 mM KCl, 2 mM MgSO_4_, 0.8 mg/ml CTAB (hexadecyltrimethylammonium bromide), 0.4 mg/ml sodium deoxycholate and 5.4 μl/ml β-mercaptoethanol). Subsequently, 600 μl of substrate solution (60 mM Na_2_HPO_4_, 40 mM NaH_2_PO_4_, 4 mg/ml o-nitrophenyl-β-D-galactosidase (ONPG) and 2.7 μl/ml β-mercaptoethanol) were added to each sample and the mixture was incubated at 30°C (until yellow color development). The reaction was stopped by the addition of 700 μl of 1M Na_2_CO_3_ and the absorbance at 420 nm was assessed spectrophotometrically after brief centrifugation. The Miller units were calculated according to the following equation [47] [48]:


$$\begin{array}{c} Miller\;units=1000\times Abs420\;nm\;/(OD600\times reaction\;time\;(minutes)\times volume\;(mL))\text{.} \end{array}$$

### Western blotting

The expression level of different RcsD periplasmic domain mutants were compared to wild-type RcsD expressed from the same plasmid using western blotting as described in (Hussein. et al., 2018) with some modifications. Briefly, 1 ml samples from actively growing cultures were mixed with trichloroacetic acid (TCA) to a final concentration of 20% followed by acetone wash. The pellet was subsequently dissolved by heating at 60°C with 1X reducing Laemmli buffer (normalized to the culture OD600). These prepared samples were then loaded onto 10% SDS-PAGE gel [49].

Subsequently, the proteins were transferred to nitrocellulose membranes (Thermo) using a transfer buffer (25 mM Tris base, 190 mM glycine, and 20% methanol in 1000 ml distilled water, adjusted to pH 8.3) (The wet transfer method was used, with a voltage of 90 volts for 120 minutes). The membranes were blocked with 5% non-fat dry milk and then probed with anti-Penta-His-HRP Conjugate IgG antibodies (Qiagen) at a dilution of 1:1500. For the loading control, anti-Glyceraldehyde 3-phosphate dehydrogenase (GAPDH) Mouse IgG1 primary antibodies were used at a concentration of 1:10,000 (Sigma) after blocking with 3% bovine serum albumin. Secondary antibodies, goat anti-Mouse IgG horseradish peroxidase-conjugate, were used at a concentration of 1:8,000 (Sigma). The membranes were developed using ECL (Thermo). Chemiluminescence signals were captured using the UVP ChemStudio (Analytik Jena). The quantification of Protein Bands of the Western blot was done using ImageJ.

### Statistical analysis

Data shown represent an average of at least three biological replicates. Calculations and statistical analyses were carried out using the software GraphPad Prism 9 (GraphPad Software, Inc.). Significance testing was conducted utilizing two-sample t-tests and one-way ANOVA tests, followed by post-hoc (Tukey) analysis when necessary.

### Significance

Signaling in Gram-negative bacteria, particularly the family Enterobacteriaceae, rely on sophisticated signaling networks to sense and respond to a wide range of environmental stressors. Some signaling cascades, including the Rcs system, integrate several auxiliary protein domains, not directly involved in the core signaling cascade and their implication in signal transmission is poorly understood. Here, we show that two auxillary proteins, RcsF (an outer membrane lipoprotein and the stress sensor of the Rcs system) and RcsD (an inner membrane phosphotransfer protein) co-evolved across the family Enterobacteriaceae and are functionally correlated, despite occupying distinct subcellular compartments.

Our findings highlight the dual advantage of two proteins with different subcellular localizations to ensure regulated stress sensing and signal transfer.

## Supporting information

S1 Fig Mantel correlation analysis between RcsC, RcsF, and the reference species phylogeny. (A) Correlation between the reference GTDB species phylogeny and the RcsC protein phylogeny, shown as a scatterplot of pairwise patristic distances (Mantel test r = 0.895, p = 0.0001). (B) Correlation between the reference GTDB species phylogeny and the RcsF protein phylogeny (Mantel test r = 0.902, p = 0.0001). (C) Correlation between the RcsC and RcsF protein phylogenies (Mantel test r = 0.958, p = 0.0001). After controlling for the species tree, the RcsC–RcsF relationship remained significant but was reduced (partial Mantel test r = 0.785, p = 0.0001), indicating that their congruence is strongly influenced by shared species history. Each point represents a pairwise species comparison; regression lines are shown for visualization.
(DOCX)

S2 Fig Structural visualization of selected residues in the periplasmic domain of RcsD. The periplasmic domain of RcsD is shown as a transparent model to facilitate visualization of the highlighted residues. (**A**) Pro222, Gly223, Asp253, Ala254, and Lys171 are shown in stick representation. (**B**) Asp48, Tyr51, and Asn140 are highlighted. Residues are displayed to emphasize their spatial distribution within the periplasmic domain.
(DOCX)

S3 Fig Structural superposition of the experimental and HADDOCK-modeled RcsF–IgaA complexes. The experimental RcsF–IgaA complex (PDB: 9BIY) is shown in gray, and the HADDOCK-modeled complex is shown with IgaA in orange and RcsF in blue. The complexes were superposed based on 259 Cα atoms of IgaA, yielding an RMSD of 0.349 Å. Using a 5 Å interface cutoff, 13 of 21 experimental IgaA interface residues were also present at the modeled interface. However, RcsF adopts a different orientation and contact interface in the HADDOCK model.
(DOCX)

S4 Fig A-Protein expression level of wild-type RcsD and RcsD periplasmic domain mutants using western blotting showing the constructs’ bands and GAPDH as the loading control. **B**- Protein expression level of wild-type RcsD and Free RcsD periplasmic.
(DOCX)

S5 Fig Normalized protein expression levels (the intensity of bands corresponding to RcsD variants detected by Penta-His-HRP antibody were normalized to the intensity of bands corresponding to GAPDH). (DOCX)

S1 Table Species from the Enterobacteriaceae family used to construct te phylogentic tree. (XLSX)

S2 Table Plasmids used and generated in this study. (XLSX)

S3 Table List of E.coli strains used and generated in this study. (XLSX)
