# Supplementary material for: Co-evolution of RcsD and RcsF shapes signal transduction in the enterobacteriaceae Rcs system
Source: PLoS One. 2026 Sep 25;21(9):e0358890. doi: 10.1371/journal.pone.0358890 (PMC13614576; doi:10.1371/journal.pone.0358890)
Supplement: Supplementary file 1 [file pone.0358890.s001.docx]

**S1 Fig. Mantel correlation analysis between RcsC, RcsF, and the reference species phylogeny.**
(A) Correlation between the reference GTDB species phylogeny and the RcsC protein phylogeny, shown as a scatterplot of pairwise patristic distances (Mantel test r = 0.895, p = 0.0001). (B) Correlation between the reference GTDB species phylogeny and the RcsF protein phylogeny (Mantel test r = 0.902, p = 0.0001). (C) Correlation between the RcsC and RcsF protein phylogenies (Mantel test r = 0.958, p = 0.0001). After controlling for the species tree, the RcsC–RcsF relationship remained significant but was reduced (partial Mantel test r = 0.785, p = 0.0001), indicating that their congruence is strongly influenced by shared species history. Each point represents a pairwise species comparison; regression lines are shown for visualization.
