# Supplementary material for: Co-evolution of RcsD and RcsF shapes signal transduction in the enterobacteriaceae Rcs system
Source: PLoS One. 2026 Sep 25;21(9):e0358890. doi: 10.1371/journal.pone.0358890 (PMC13614576; doi:10.1371/journal.pone.0358890)
Supplement: Supplementary file 2 [file pone.0358890.s002.docx]

**S2 Fig. Structural visualization of selected residues in the periplasmic domain of RcsD.**
The periplasmic domain of RcsD is shown as a transparent model to facilitate visualization of the highlighted residues. (A) Pro222, Gly223, Asp253, Ala254, and Lys171 are shown in stick representation. (B) Asp48, Tyr51, and Asn140 are highlighted. Residues are displayed to emphasize their spatial distribution within the periplasmic domain.
