# Supplementary material for: Co-evolution of RcsD and RcsF shapes signal transduction in the enterobacteriaceae Rcs system
Source: PLoS One. 2026 Sep 25;21(9):e0358890. doi: 10.1371/journal.pone.0358890 (PMC13614576; doi:10.1371/journal.pone.0358890)
Supplement: Supplementary file 3 [file pone.0358890.s003.docx]

**S3 Fig. Structural superposition of the experimental and HADDOCK-modeled RcsF–IgaA complexes.**
The experimental RcsF–IgaA complex is shown in gray, and the HADDOCK-modeled complex is shown with IgaA in orange and RcsF in blue. The structures were superposed with an RMSD of 0.391 Å.
