# Supplementary material for: Co-evolution of RcsD and RcsF shapes signal transduction in the enterobacteriaceae Rcs system
Source: PLoS One. 2026 Sep 25;21(9):e0358890. doi: 10.1371/journal.pone.0358890 (PMC13614576; doi:10.1371/journal.pone.0358890)
Supplement: Supplementary file 4 [file pone.0358890.s004.docx]

S4 Fig **A**-Protein expression level of wild-type RcsD and RcsD periplasmic domain mutants using western blotting showing the constructs' bands and GAPDH as the loading control. **B**- Protein expression level of wild-type RcsD and Free RcsD periplasmic.
