# Supplementary material for: Co-evolution of RcsD and RcsF shapes signal transduction in the enterobacteriaceae Rcs system
Source: PLoS One. 2026 Sep 25;21(9):e0358890. doi: 10.1371/journal.pone.0358890 (PMC13614576; doi:10.1371/journal.pone.0358890)
Supplement: Supplementary file 5 [file pone.0358890.s005.docx]

S5 Fig. Normalized protein expression levels (the intensity of bands corresponding to RcsD variants detected by Penta his-HRP antibody were normalized to the intensity of bands corresponding to GAPDH).
